# Elevated serum visfatin levels are associated with poor prognosis of hepatocellular carcinoma

**DOI:** 10.18632/oncotarget.15080

**Published:** 2017-02-04

**Authors:** Yifan Sun, Shengbo Zhu, Zhitong Wu, Yiyong Huang, Chunming Liu, Shifu Tang, Lili Wei

**Affiliations:** ^1^ Department of Clinical Laboratory, Affiliated Liutie Central Hospital of Guangxi Medical University, Liuzhou, Guangxi, China; ^2^ Department of Clinical Laboratory, Third Affiliated Hospital of Guangxi University of Chinese Medicine, Liuzhou, Guangxi, China; ^3^ Department of Clinical Laboratory, Eighth Affiliated Hospital of Guangxi Medical University, Guigang City People's Hospital, Guigang, Guangxi, China; ^4^ Department of Science and Education, Third Affiliated Hospital of Guangxi University of Chinese Medicine, Liuzhou, Guangxi, China

**Keywords:** serum, visfatin, hepatocellular carcinoma, prognosis

## Abstract

Visfatin is considered a pro-inflammatory adipocytokine, and it is commonly increased in obesity-related diseases. This study aimed to evaluate the levels of serum visfatin in patients with hepatocellular carcinoma (HCC) and its diagnostic and predictive value in detecting HCC. Fasting serum levels of visfatin of 135 HCC patients, 115 chronic hepatitis B (CHB) patients, 129 liver cirrhosis (LC) patients, and 149 healthy controls were determined via enzyme-linked immunosorbent assay. Meanwhile, serum alpha fetal protein (AFP) and interleukin-6 (IL-6) were also assayed. The median serum visfatin concentration in HCC patients was 1.113 ng/mL (range: 0.823-2.214 ng/mL), which was significant higher than those of healthy controls, CHB patients, and LC patients (*P*<0.05). The serum visfatin concentration in HCC patients was positively correlated with AFP (r=0.595, *P*<0.001) and IL-6 (r=0.261, *P*<0.015) and was also associated with tumor size and tumor node metastasis stage. Moreover, elevated levels of serum visfatin were associated with a higher HCC risk for CHB and LC patients. Multivariate Cox regression analysis had shown that HCC patients with high levels of serum visfatin had significantly shorter overall survival times than those with low serum visfatin levels (*P*<0.001). Using a cutoff visfatin level of 1.403 ng/mL, the receiver operating characteristic curve analysis showed unappealing sensitivity and specificity values (45.76% and 74.79%, respectively; AUC=0.626) regarding visfatin's use as a diagnostic marker for HCC. Our results indicate that increased serum visfatin levels are associated with poor prognosis of HCC. Visfatin may be a potential therapeutic target of HCC.

## INTRODUCTION

Hepatocellular carcinoma (HCC) is a common type of liver cancer with high morbidity and mortality, and it is the third most frequent cause of cancer-related deaths, moreover, the number of new cases of HCC is increasing year by year [1]. Hepatitis B (HBV) and C (HCV) infections, aflatoxin-B1 (AFB1) exposure, and excessive alcohol intake are commonly recognized etiologies linked to the development of HCC [1, 2]. However, HCC is a multifactorial disease, and the accurate etiology of HCC remains elusive. Over the last two decades, the rising incidence of HCC has been related to the burgeoning incidence of obesity, nonalcoholic fatty liver disease (NAFLD), and metabolic syndrome [3].

There is sufficient evidence that obesity is an important cause of many forms of cancer [4–6]. Mechanisms that link obesity and cancer risk are mainly focused on three hormonal systems: the insulin and insulin-like growth factor (IGF) axis, sex steroids, and adipokines [6]. Visfatin — also called nicotinamide phosphoribosyltransferase (NAMPT), or pre-B-cell colony-enhancing factor (PBEF) — is the most abundant adipokine secreted mainly from the visceral fat adipocytes, and it is positively correlated with body mass index (BMI). Visfatin is upregulated during adipocyte differentiation and has insulin-like activity under physiological conditions; hence, elevated visfatin levels have been found to be positive correlated with metabolic syndromes, such as polycystic ovary syndrome [7], type 2 diabetes mellitus (T2DM) [8], and cardiovascular diseases [9]. In addition, visfatin is also one of the inflammatory cytokines and can interact with interleukin-6 (IL-6) and tumor necrosis factor-alpha (TNF-α)[10]. Moreover, visfatin has the ability to regulate a variety of signaling pathways that play key roles in the pathologic process of cancers, such as PI3K/Akt, ERK1/2, and STAT3 [11]. Therefore, elevated visfatin expression may impact the development of various cancers and could be a potential therapeutic target in the effective control of cancers.

Previous studies have identified that circulating visfatin levels are elevated in patients with colorectal cancer [12], gastric cancer [13], breast cancer [14], prostate cancer [15], pancreatic cancer [16], and oral cancers [17]. Ninomiya et al. [18] have found a positive correlation between HCC tumor size and serum visfatin levels, regardless of complications related to obesity and diabetes. However, the levels of circulating visfatin in HCC patients and their value in the diagnosis and prognosis of HCC remain unclear. In this study, we performed circulating visfatin expression analysis comprehensively to investigate the potential association between serum visfatin levels and the characteristics of HCC using a retrospective study.

## RESULTS

### Serum visfatin levels in HCC patients

**Figure 1:**
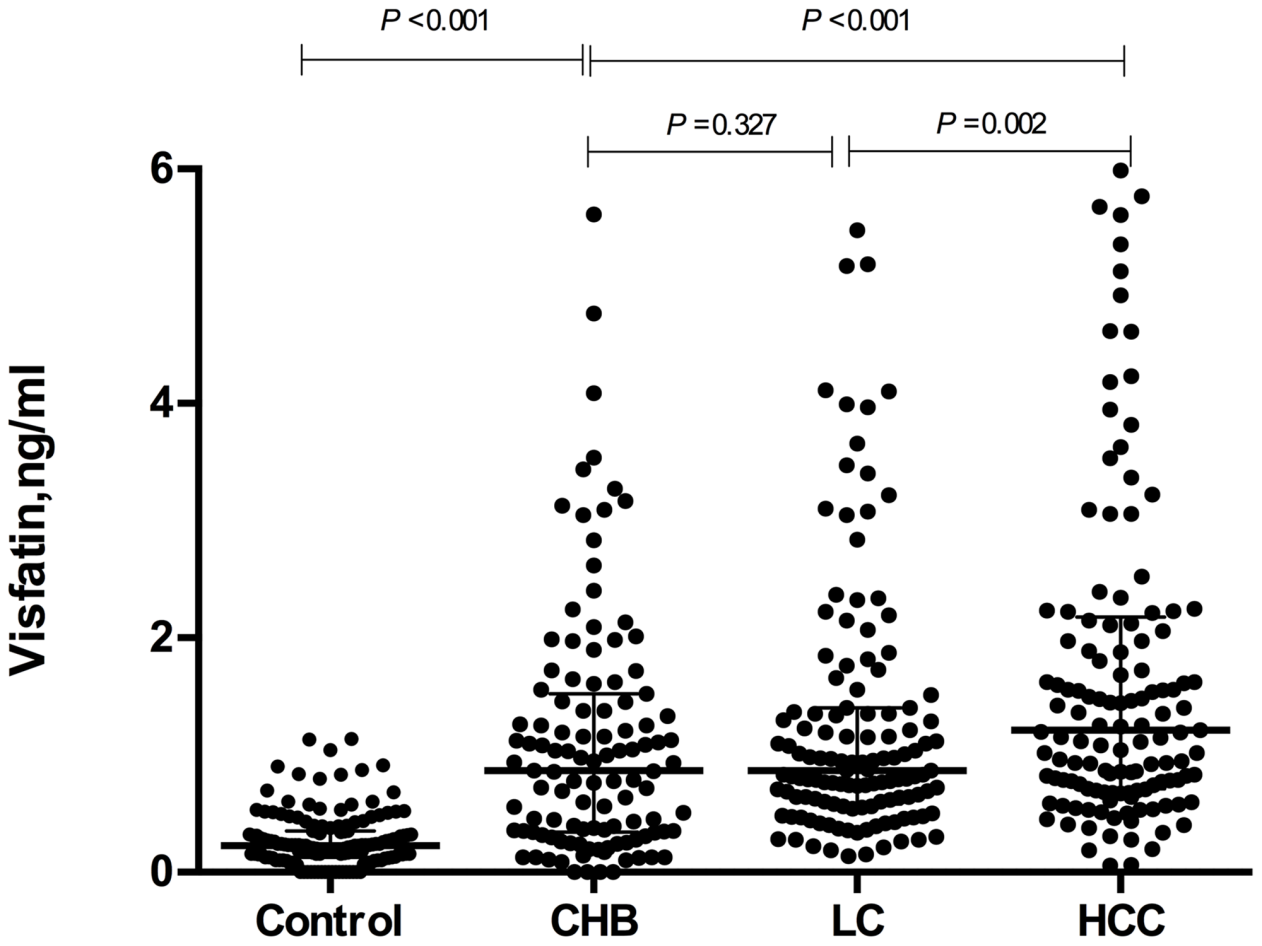
The serum visfatin concentration in liver diseases and controls CHB, chronic hepatitis B; LC, liver cirrhosis; HCC, hepatocellular carcinoma.

The basic characteristics and clinical laboratory parameters of the study population are shown in Table 1. Significant differences were found between the patients and controls in terms of gender and age (*P* < 0.05) but not smoking, drinking, and BMI. In the test of the laboratory parameters, such as AFP, TP, ALB, ALT, and AST, significant differences were also observed between each group (*P*< 0.001). With respect to serum visfatin levels, the median serum visfatin concentration in HCC patients was 1.113 ng/mL (range: 0.823-2.214 ng/mL), which was significant higher than those of healthy controls, CHB patients, and LC patients (P<0.05, Table 1 and Figure 1). The levels of serum visfatin were also significantly increased in CHB and LC patients as compared with controls (*P*< 0.001); however, there was no significant difference in serum visfatin levels in the two groups (*P*=0.327, Figure 1).

**Table 1:** The characteristics of study population

Characteristics	Control (N=149)	CHB(N=115)	LC(N=129)	HCC(N=135)	*P*
Age (years)	46.34±7.12	45.27±9.768	48.71±11.65	49.93±11.72	0.001*
Gender					<0.001^▴^
Man	54.4(81)	66.1(76)	77.5(100)	87.4(118)	
Female	45.6(68)	33.9(39)	22.5(29)	12.6(17)	
Smoking					0.108^▴^
Yes	30.9(46)	42.6(49)	38.8(50)	30.4(41)	
No	69.1(103)	57.4(66)	61.2(79)	69.6(94)	
Drinking					0.703^▴^
Yes	28.2(42)	34.8(40)	32.6(42)	31.1(42)	
No	71.8(107)	65.2(75)	67.4(87)	68.9(93)	
BMI (kg/m^2^)	22.34±3.55	21.94±3.56	22.86±3.89	22.06±3.31	0.173*
AFP (ng/mL)	-	12.7(3.6-65.2)	8.0(3.0-18.7)	324.9(9.9-6128.3)	<0.001^#^
TP (g/L)	74.0±6.3	71.6±8.8	71.5±9.5	68.0±9.2	<0.001*
ALB (g/L)	41.4±5.1	40.8±6.8	37.0±8.4	38.0±5.5	<0.001*
ALT (IU/L)	22(18-30)	47(28-78)	43(28-43)	47(31-86)	<0.001^#^
AST (IU/L)	22(15-31)	39(28-86)	33(23-47)	42(29-78)	<0.001^#^
IL-6 (mmol/L)	-	-	-	65.32±31.63	
Visfatin (ng/mL)	0.759(0.714-0.810)	0.868(0.345-1.521)	0.919(0.743-1.476)	1.113(0.823-2.214)	<0.001^#^

* a one-way ANOVA test; ^▴^ *χ^2^* test; ^#^Kruskal-Wallis test.

The associations between various serum visfatin levels and HCC risk were analyzed. According to the serum visfatin concentration in CHB and LC patients, the quartile intervals for the serum visfatin levels were <0.51 ng/mL, 0.51-0.89 ng/mL, 0.891-1.454 ng/mL, and ≥1.455 ng/mL. The frequency of serum visfatin levels ≥1.455 ng/mL in HCC patients was significant higher than that of CHB+LC patients (31.8% vs 25.0%). When compared with serum visfatin levels <0.51 ng/mL (Table 2), HCC risk was significantly increased for 0.51-0.89 ng/mL levels (OR=5.34, 95CI%=2.050-13.894), 0.891-1.454 ng/mL levels (OR=4.90, 95CI%=1.914-12.526), and ≥1.455 ng/mL levels (OR=6.69, 95CI%=2.648-16.879), indicating there was a high HCC risk in CHB and LC patients with elevated serum visfatin levels.

**Table 2:** The associations between various serum visfatin levels and HCC risk

Quartile intervals (ng/mL)	HCC		CHB+LC		OR	95%CI
	N	Frequency(%)	N	Frequency(%)		
<0.51	6	4.4	61	25	1^ref^	
0.51-0.89	38	28.1	61	25	5.34	2.050-13.894
0.891-1.454	40	29.6	61	25	4.90	1.914-12.526
≥1.455	51	37.8	61	25	6.69	2.648-16.879

**Figure 2:**
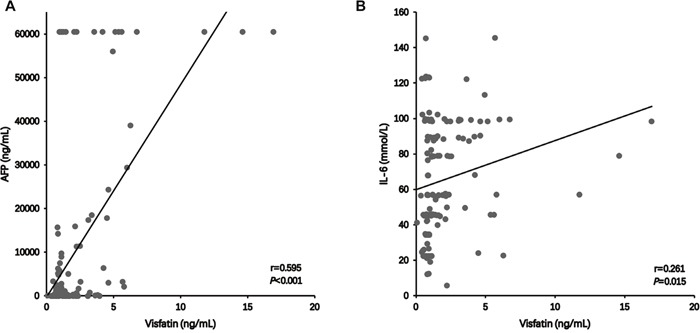
The correlation between serum visfatin and AFP **A**., IL-6 **B**. concentration in patients with HCC.

The possible association between serum visfatin concentration and AFP and IL-6 in HCC patients was analyzed further. In 135 HCC patients, the serum AFP and IL-6 concentrations were 324.9 ng/mL (range: 9.9-6128.3 ng/mL) and 65.32 ± 31.63 mmol/L, respectively. The mean Pearson product-moment correlation coefficient (*r*) of AFP with serum visfatin concentration was 0.595 (*P*<0.001, Figure 2A), indicating a positive correlation between serum AFP and visfatin concentration in HCC patients. Similar results (*r*=0.261, P=0.015, Figure 2B) were also obtained between the serum IL-6 and visfatin concentrations in HCC patients.

**Figure 3:**
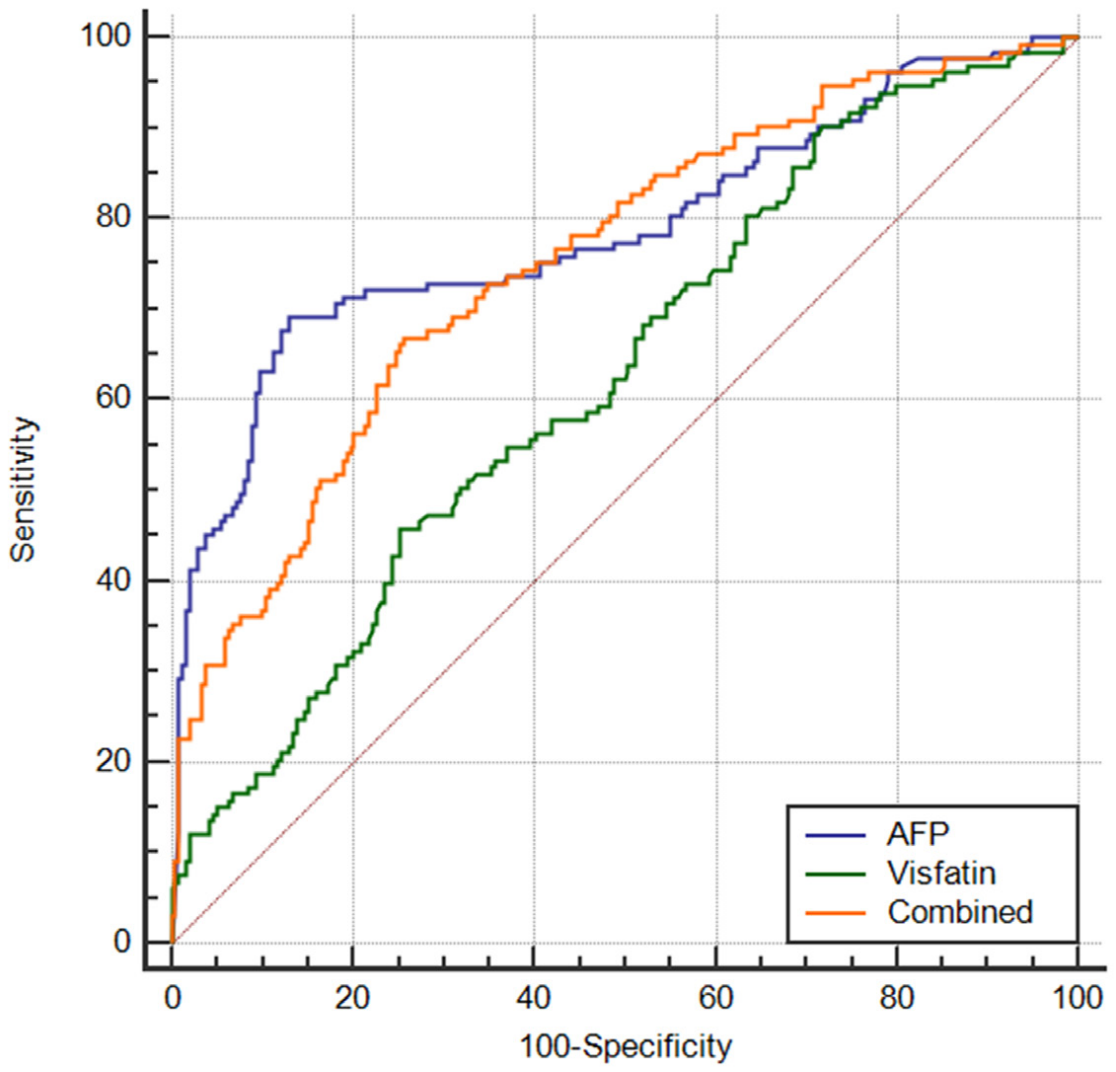
Receiver operating characteristic curve analysis for the predictive performance of serum visfatin for HCC

To evaluate the diagnostic accuracy of serum visfatin levels between HCC patients and CHB+LC subjects, ROC curve analysis was performed (Figure 3). The AUC of the ROC curve of serum visfatin, when used to predict HCC, was 0.626 (95%CI=0.575-0.676). The best cutoff value was 1.403 ng/mL, with a sensitivity of 45.76% and a specificity of 74.79%. In comparison, the AUC of AFP to predict HCC was 0.788 (95%CI=0.743-0.828, AUC_AFP_ vs AUC_visfatin_: *P*<0.001), with a sensitivity of 69.17% and a specificity of 86.97%. The AUC for the combination of visfatin and AFP was 0.753(95%CI=0.706-0.796), which was similar to that of AFP(*P*=0.296).

### Relationship between serum visfatin levels and clinical factors in HCC patients

According to the best cutoff value detected via ROC curve analysis, serum visfatin levels were bifurcated at 1.403 ng/mL. Values ≥1.403 ng/mL were considered high levels of serum visfatin, and values <1.403 ng/mL indicated low levels of serum visfatin. As show in Table 3, it was found that high serum visfatin levels were significantly correlated with gender, smoking, tumor size, tumor stage, and 5-year survival (P<0.05).

**Table 3:** Relationship between serum visfatin levels and clinical factors in HCC patients

Characteristics	Low Visfatin level	High Visfatin level	χ2	P
	(n=81)	(n=54)		
Age			0.028	0.867
<60	62(76.5)	42(77.8)		
>60	19(23.5)	12(22.2)		
Gender			4.049	0.044
Man	67(82.7)	51(94.4)		
Female	14(17.3)	3(5.6)		
Smoking			4.256	0.039
Yes	30(37.0)	11(20.4)		
No	51(63.0)	43(79.6)		
Drinking			0.092	0.761
Yes	26(32.1)	16(29.6)		
No	55(67.9)	38(70.4)		
Tumor size			10.709	0.001
<3cm	56(69.1)	22(40.7)		
≥3cm	25(30.9)	32(59.3)		
Tumor stage			6.058	0.014
I, II	49(60.5)	21(38.9)		
III, IV	32(39.5)	33(61.1)		
5 years survival			3.913	0.048
Dead	65(80.2)	50(92.6)		
Alive	16(19.8)	4(7.4)		

### Overall survival analysis in HCC patients

**Figure 4:**
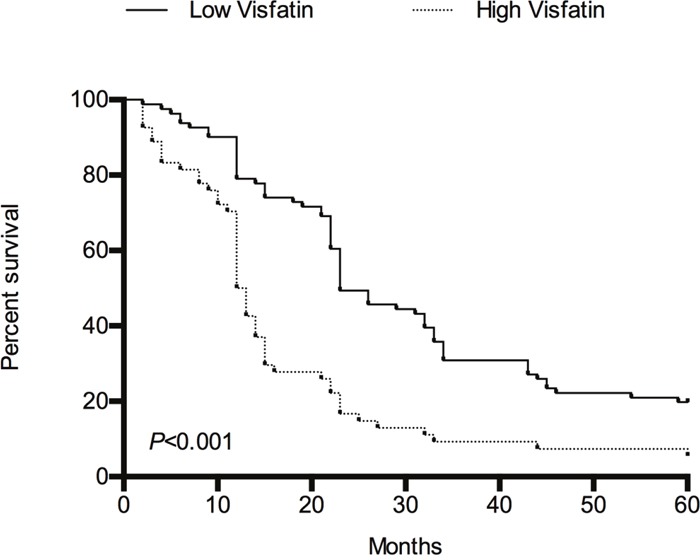
The overall survival of HCC patients stratified by serum visfatin levels during 5-year follow-up using Kaplan–Meier method

During the 5-year follow-up, the median overall survival was 23.0 months for those with low serum visfatin levels(<1.403 ng/mL) and 12.5 months for those with high serum visfatin levels(≥1.403 ng/mL). As shown in Figure 4, an analysis of the overall survival of HCC patients stratified by serum visfatin levels suggests that HCC patients with high serum visfatin levels had significantly shorter overall survival times than those with low serum visfatin levels (*P*<0.001).

## DISCUSSION

In this study, we comprehensively evaluated the potential value of serum visfatin in HCC patients, and results indicated that serum visfatin levels were significantly elevated in patients with HCC and were associated with AFP, IL-6, tumor size, and tumor stage. Meanwhile, we also found that high serum visfatin levels were correlated with high HCC risk and poor overall survival. However, the ROC analysis showed that visfatin did not have superior predictive value for HCC as compared with that of AFP.

Adipose tissue is a complex endocrine system that produces a large number of adipokines, such as leptin, resistin, adiponectin, and visfatin. These adipocytokines are commonly increased in obesity-related such as NAFLD and metabolic syndrome, including HCC, and play a fundamental role in these diseases [19]. For example, increased leptin expression has been found in HCC cell lines and HCC tissue, as well as to increase tumor invasiveness and the migration of HCC [20, 21]. In contrast, the increased adiponectin expression in HCC patients antagonizes the oncogenic actions of leptin in HCC [22]. In this study, we also observed circulating visfatin to be significantly increased in patients with liver diseases, especially HCC. Similar results have been obtained in other patients with digestion system tumors. In 358 colorectal cancer (CRC) cases and 286 controls, Chen et al.[23] found that the visfatin levels in patients with CRC were significantly higher than those in controls (P<0.05). When detecting the Nampt protein in the cytoplasm, Chen et al.[24] found that the levels of Nampt in 68 gastric carcinoma cases (13±5) were significantly higher than those in 59 normal gastric tissues (6±3) (P < 0.01). Increased visfatin expression has also been found in the plasma of 262 gastric cancer patients [25]. However, Chen et al [26] did not observed increased serum visfatin levels in HCC patients as compared to controls (11.10/12.30), which stands in contrast to our results. In a recent meta-analysis, Chang et al. [27] observed a positive correlation between elevated serum visfatin levels and overweight/obesity(BMI≥25kg/m^2^). Another study by Jurdana et al. [28] also found higher serum visfatin levels in overweight/obesity(BMI≥29kg/m^2^) subjects than in controls (4.1±0.6 ng/mL vs.1.8±0.6 ng/mL for males and 4.4±0.5 ng/mL vs. 2.5±0.5 ng/mL for females). Therefore, we selected participants with BMI<25 kg/m^2^ in this study to reduce the effect of overweight/obesity on serum visfatin levels. However, the study performed by Chen et al. [26] involved 38.2% overweight/obesity subjects among controls, which may be the main reason for the inconsistent results between our study and theirs.

The increased visfatin concentration in patients with HCC suggests that visfatin may be involved in the process of HCC. Actually, we found that high serum visfatin levels were related to HCC risk. The role of visfatin in the development of HCC has been attributed to several possible mechanisms. First, visfatin can be considered a pro-inflammatory adipocytokine. The expression of visfatin has been shown to closely interact with inflammation and immune-related cytokines; for example, visfatin induces the production of IL-1beta, TNF-α, and especially IL-6 [10], and incubation with IL-6 in HUVECs induced a significant increase in visfatin mRNA levels [25]. In this study, we also found that serum visfatin concentration in HCC patients was positively correlated with serum IL-6 concentration, indicating that visfatin plays a role in inflammatory in HCC. In fact, adipocytokines are similar to classical cytokines. For example, leptin has many pro-inflammatory functions and can increase the production of pro-inflammatory cytokines, such as IL-6, TNF-α, and IL-12 [26]. Similar to IL-6, visfatin preferentially stimulated the proliferation of HepG2, Hep3B, and HuH7 human HCC cells [18]. Secondly, the overexpression of visfatin increased the activity of a number of signaling pathways that encourage carcinogenesis, such as NAD-dependent SIRTs, PI3K/Akt, ERK1/2, and STAT3 [11, 27]. Similar to our study, Chen et al. [23] also found that patients with high levels of visfatin had a higher risk for early and advanced CRC (OR=3.37, *P*=0.011; OR= 2.38, *P*=0.015, respectively). Therefore, decreasing visfatin expression might be a potential strategy for the prevention of HCC in chronic liver diseases.

Furthermore, we found that high levels of serum visfatin were significantly associated with larger tumor size and higher tumor stage. Consistent with our results, Ninomiya et al.[18] found that serum visfatin concentration in HCC patients was positively correlated with tumor size. In another study, elevated plasma visfatin levels in gastric cancer patients was found to be correlated with invasion depth, lymph node metastasis, distant metastasis, peritoneal dissemination, tumor size, and tumor node metastasis stage; furthermore, multivariate Cox regression analysis identified plasma visfatin level as an independent predictor of overall survival [28]. In this study, we also found that HCC patients with high serum visfatin levels had significantly shorter overall survival times than those with low serum visfatin levels (*P*<0.001). These results suggest that elevated visfatin expression can accelerate the progress of cancers. Indeed, *in vitro*, visfatin leads to increased cancer cell survival and migration [29], the preferentially stimulated proliferation of HCC cells [18], and the increased viability of the cancer cells by inducing antioxidative activity [30]. *In vivo*, the visfatin levels decreased significantly after chemotherapy [31]. On the basis of previous studies and our results, visfatin could be a potential therapeutic target of HCC.

Although elevated levels of visfatin have been observed in various cancers, very few studies have investigated the diagnostic and predictive value of serum visfatin for detecting cancer. Some studies have shown acceptable diagnostic value on the part of circulating visfatin levels for the detection of early CRC (sensitivity= 68.2% and specificity=70.8%, AUC=0.812)[32] and postmenopausal breast cancer (sensitivity= 97.6% and specificity=92.6%, AUC=0.724)[33]. To our knowledge, this is the first study to investigate the potential diagnostic value of serum visfatin levels for HCC. In this study, with respect to serum visfatin, ROC analysis showed unappealing sensitivity and specificity values regarding diagnostic markers for HCC, especially compared with those of AFP, a traditional tumor marker for HCC, although the serum visfatin concentration in HCC patients was positively correlated with AFP concentration. Therefore, we do not recommend adding visfatin to the set of diagnostic biomarkers for HCC.

Our study has a few limitations. Visfatin, as a form of extracellular NAMPT, is susceptible to overexpression under oxidative stress in HCC cells; it then inhibits the growth of hepatoma cells [34]. In contrast, Buldak et al. [30] observed that elevated visfatin levels induced antioxidant capacity and promoted melanoma cell proliferation. Therefore, visfatin may play different roles in the development of HCC, and monitoring serum visfatin levels in various HCC stages will strengthen our conclusions. Additionally, only participants with BMI<25 kg/m^2^ were included in this study, therefore, the role of visfatin in HCC in obese patients remains unclear; in addition, the relation between visfatin levels and the other index of of visceral fat mass, such as waist circumference, waist-hip ratio, lipid accumulation product, are not evaluated and adjusted in this study, which might produce potential bias for our results, as visfatin is upregulated during adipocyte differentiation. Finally, this study only focused on the patients with HBV infection, the role of visfatin levels in HCC patients with HCV, alcohol, and autoimmune hepatitis, should be further studied in future.

In summary, our data found, for the first time, significantly increased serum visfatin levels to be associated with higher HCC risk, higher-grade malignancy, and poor overall survival. The results of the present study indicate that targeting visfatin might be a promising strategy for the prevention or treatment of HCC patients.

## MATERIALS AND METHODS

### Subjects

All participants were recruited at the Third Affiliated Hospital of Guangxi University of Chinese Medicine in Liuzhou, China, from April 2010 to January 2011. All patients were hepatitis-B-surface-antigen- and hepatitis-B-virus-core-antibody-positive. The diagnostic criteria for chronic hepatitis B (CHB) included the following: elevated serum alanine aminotransferase (ALT) or aspartate aminotransferase (AST) levels (> 40 IU/mL) or HBV-DNA levels > 1,000 copies/mL. The liver cirrhosis (LC) patients were diagnosed on the basis of a pathological examination, laboratory features, and the findings of computed tomography (CT) or ultrasonography. The HCC patients were diagnosed based on a continued rise in serum alpha fetal protein (AFP) levels (> 400 ng/mL) and an imaging examination or confirmed via pathological examination; these patients underwent surgery. All HCC patients were followed up every 3 months for 3 years and thereafter every 6 months for 2 years. Finally, 115 CHB patients, 129 LC patients, and 135 HCC patients were included in this study. In addition, 149 healthy people were selected as controls.

The exclusion criteria were co-infection with hepatitis A/C/D/E viruses and having other liver diseases, such as alcoholic liver diseases or autoimmune hepatitis. To avoid the effect of obesity on visfatin levels, patients with BMI≥25kg/m^2^ were excluded.

All participants provided written informed consent for this study, and this study was approved by the Ethics Committee of the Third Affiliated Hospital of Guangxi University of Chinese Medicine.

### Immunoassay methods

Fasting peripheral venous blood was collected from all participants at the time of diagnosis, and after centrifugation for 10 minutes at 3000g, the serum samples were stored at -80°C prior to assay. Serum levels of visfatin were determined via enzyme-linked immunosorbent assay (CUSABIO BIOTECH); the intra-assay precision was < 8.0%, and the inter-assay precision was < 10.0%. Serum alpha fetal protein (AFP) and IL-6 concentrations were measured with the Cobas e601 system.

### Statistical analysis

Continuous variables were expressed as means±standard deviations (SD) if they were normally distributed upon Shapiro-Wilk test, and differences in the data were compared with a one-way ANOVA test; otherwise, data were shown as the median and interquartile range (IRQ) and were tested via Kruskal-Wallis test. To avoid the false positive results, the extremely high data (>3SD) were excluded when compared. Differences in qualitative characteristics, such as the distributions of gender, ethnicity, smoking, drinking, tumor stage, and tumor size, were analyzed using the Chi-squared test. In addition, the degrees of association between continuous variables were calculated via Pearson correlation coefficient. The predictive performance of visfatin levels for HCC was evaluated using a receiver operating characteristic (ROC) curve analysis, along with the calculated area under the curve (AUC). The sensitivities and specificities were calculated using a cut-off value that was selected from the ROC curve. Odds ratios (ORs) with 95% confidence intervals (CIs) were calculated for different degrees of serum visfatin in both HCC and CHB+LC groups, using a binary logistic regression analysis by adjusting for confounding factors, such as age, gender, smoking, and drinking. Overall survival was estimated using the Kaplan–Meier method, and intergroup differences in survival time were tested using the log-rank test.

The statistical analysis was performed using SPSS 19.0 software (IBM Corporation, Armonk, NY, USA), GraphPad Prism 6.0 (GraphPad Software, La Jolla, CA, USA), and MedCalc 15.6.1 software (MedCalc Software bvba, Ostend, Belgium) as appropriate. All statistical tests were two-tailed, and a *P*-value < 0.05 was considered statistically significant.
